# Myasthenia gravis associated with renal cell carcinoma: a paraneoplastic syndrome or just a coincidence

**DOI:** 10.1186/s12883-021-02311-8

**Published:** 2021-07-12

**Authors:** Yiming Zheng, Jingjing Luo, Haiqiang Jin, Ran Liu, Hongjun Hao, Feng Gao

**Affiliations:** Neurology Department, Peking University First Hospital, No.8 Xishiku Street, Beijing, 100034 China

## Abstract

**Background:**

Myasthenia gravis (MG) can occur as a paraneoplastic phenomenon associated with thymoma. The association of MG with renal cell carcinoma (RCC) is not clear. Herein, we describe six cases of MG associated with RCC.

**Methods:**

There were 283 patients diagnosed with MG admitted to our hospital from 2014 to 2019. Among them, 6 patients also had RCC. None of them had immune checkpoint inhibitor therapies. We performed a retrospective clinical data collection and follow-up studies of these 6 patients.

**Results:**

These 6 patients with an average MG onset age of 61.3 ± 13.3 years, were all positive for anti-acetylcholine receptor antibodies. MG symptoms appeared after RCC resection in 3 cases. RCC was discovered after the onset of MG in 2 cases, and synchronously with MG in 1 case. After nephrectomy, the MG symptoms showed a stable complete remission in 1 case. Among them, four patients met the diagnostic criteria of possible paraneoplastic neurological syndromes.

**Conclusions:**

Except for thymoma, patients with MG should pay attention to other tumors including RCC. MG may be a paraneoplastic syndrome of RCC, and further studies are needed to elucidate the relationship.

**Supplementary Information:**

The online version contains supplementary material available at 10.1186/s12883-021-02311-8.

## Background

Myasthenia gravis (MG), an autoimmune neuromuscular disease caused by autoantibodies mostly against nicotinic acetylcholine receptors (AChRs) in neuromuscular junctions, can occur as a paraneoplastic phenomenon associated with thymoma [1, 2]. The association between extrathymic malignancies and MG is an attractive topic. Several cases of extrathymic malignancies complicated with MG have been reported, considering as paraneoplastic syndromes. Most of the reported extrathymic malignancies are lung cancer [3–7]. As far as we known, there is only one report that MG as a paraneoplastic syndrome associated with renal cell carcinoma (RCC) in the medical literature [7]. To date, there has been no more evidence supporting the speculation that association of MG with RCC might be a paraneoplastic syndrome or just a coincidence. Herein, we describe six cases of MG associated with RCC.

## Methods

There were 283 patients diagnosed with MG admitted to our hospital from 2014 to 2019 by searching the electronic medical record system. These patients all fulfilled the diagnostic criteria for MG. These patients were including 127 males and 156 females (Male to female, 1:1.2), with a mean age of 58.2 ± 17.2 years (16–90 years) and an average MG onset age of 54.9 ± 18.6 years (4–89 years). The MGFA clinical classification classes were including type I (50 patients), type IIa (31 patients), type IIb (16 patients), type IIIa (30 patients), type IIIb (80 patients), type IVa (13 patients), type IVb (34 patients) and type V (29 patients). There were 23.0% patients with thymoma. The presence of anti-AChRs antibodies was 60.6%. Among them, 6 patients also had RCC diagnosed by pathological examination. None of them had immune checkpoint inhibitor therapies. We performed a retrospective clinical data collection and follow-up studies of these 6 cases of MG associated with RCC. Written informed consent was obtained from all participants or their legal surrogate. Research Ethics Committee of The First Hospital of Peking University approved this study.

## Results

These 6 patients were including 5 males and 1 female, with an average MG onset age of 61.3 ± 13.3 years (39–76 years). Among them, 5 patients had the onset of ptosis, and the other one had the onset of weakness of the proximal limbs. The MGFA clinical classification classes were including one each for I, IIa, IIb, and IIIb and two for IVb. Only one patient had a thymoma. They all had anti-AChRs antibodies. The deltoid, abductor digiti minimi and orbicularis oculi muscles were tested for repetitive nerve stimulation tests. Four patients showed a positive result with a significant decrement at low-frequency stimulations, but all were negative at high-frequency stimulations. Single fiber electromyography was not performed in these patients. RCC was discovered 7 years (case 1) and 4 years (case 2) after the onset of MG in two cases. The other three cases in whom MG symptoms appeared after RCC resection for 1 month (case 3), 5 months (case 4) and 6 years (case 5, with bone metastasis). In the remaining one case (case 6), RCC was discovered by chance when the tumor was screened at the onset of MG. After radical nephrectomy, the symptoms of MG showed a stable complete remission in one case. However, the other five patients who were also treated with immunosuppressants had different outcomes (three with partially improved and two remained unchanged). The detail clinical data of these six patients were showed in the Supplementary material and summarized in Table 1. According to the recommended diagnostic criteria for paraneoplastic neurological syndromes proposed by F Graus et al., case 1 and case 2 did not meet the possible diagnosis of paraneoplastic neurological syndromes. The other four patients (case 3 to case 6) met the diagnostic criteria for possible paraneoplastic neurological syndromes [8].

**Table 1:** The clinical data of these six MG patients with RCC

	Age/sex	MG onset Age	Initial symptoms	MGFA class	Thymoma	Anti-AChR	Anti-titin	Anti-RyR	Anti-MUSK	Presenting symptoms of RCC	Sequence of MG and RCC	Histology, grading and size of RCC	Drugs for MG	Follow-up
1	83/M	71	Ptosis and diplopia	I	-	+	+	+	-	Renal dysfunction	RCC was detected 7 years after MG onset	Clear cell and chromophobe, G1, 4.1 cm*3.5 cm*3 cm	Pyridostigmine, prednisolone and azathioprine	Unchanged, stable, occasional ptosis after radical nephrectomy
2	76/F	68	Ptosis	IVb	-	+	-	-	-	Asymptomatic	RCC was detected 4 years after MG onset	Clear cell, G1, 4.1 cm*3.1 cm*4.2 cm	Pyridostigmine, prednisolone, tacrolimus and IVIg	Unchanged after radiofrequency therapy for RCC
3	59/M	57	Ptosis and diplopia	IIB	-	+	-	-	-	Hematuria	MG developed 1 month after radical nephrectomy	Clear cell, G1, 8.5 cm*7.5 cm*7 cm	Pyridostigmine	Complete stable remission for one year
4	44/M	39	Limb weakness	IIa	+	+	-	-	-	Asymptomatic	MG developed 5 months after partial nephrectomy and 1 months after thymectomy	Clear cell, G1 and G2, 3.3 cm*3 cm*2.7 cm	Pyridostigmine and prednisolone	Improved, stable, mild weakness
5	62/M	57	Ptosis and diplopia	IIIb	-	+	+	+	-	Flank pain	MG developed 6 years after radical nephrectomy	Clear cell, G1, 8.5 cm*6.5 cm*4 cm	Pyridostigmine, prednisolone and IVIg	Improved, but **died of RCC metastasis at the age of 62**
6	77/M	76	Ptosis	IVb	-	+	NA	NA	NA	Asymptomatic	synchronous	Clear cell, G1, 5 cm*4.2 cm*4 cm	Pyridostigmine, tacrolimus/mycophenolate mofetil, and IVIg	Improved; fluctuated, persistent ptosis

*Abbreviations*: *MG* Myasthenia gravis, *RCC* Renal cell carcinoma, *M* Male, *F* Female, *AChR* Aacetylcholine receptor, *RyR* Ryanodine receptor, *MUSK* Muscle-specific tyrosine kinase, *NA* Not available, *IVIg* Intravenous immune globulin

## Discussion

All these six patients were presented with typical clinical pattern of myasthenic weakness of the ocular muscles, bulbar muscle or proximal limb muscles, and positive for anti-AChRs antibodies. The diagnosis was MG definite in these six patients [1]. All these patients had pathological diagnosis with RCC, most of which were clear cell type.

There are two types of association between RCC and MG in these six cases. One is the occurrence of RCC in MG patients after many years of treatment that can be a coincidental finding (case 1 and case 2). Case 1 and case 2 did not meet the possible diagnosis of paraneoplastic neurological syndromes, because RCC was discovered more than 2 years after the onset of MG [8]. It is difficult to establish a relationship between these two diseases in these cases. Another type is the presentation of RCC within two years of the diagnosis of MG. There are three cases in whom MG symptoms appeared after RCC resection (case 3 for 1 month, case 4 for 5 months, and case 5 with bone metastasis for 6 years). In case 6, RCC was discovered at the onset of MG. Thus, these four patients (case 3 to case 6) met the diagnostic criteria for possible paraneoplastic neurological syndromes [8]. After radical nephrectomy, the symptoms of MG showed a stable complete remission without the application of immunosuppressants in case 3, which is more suggestive of a paraneoplastic association [8, 9]. However, other patients who were also treated with immunosuppressants at the same time had different outcomes. It is difficult to judge whether MG is a paraneoplastic syndrome of RCC through the prognosis in these patients. Because some patients with thymoma-associated MG can only get partial improvement or remain unchanged after thymectomy who need further pharmacological treatment. At the same time, some patients can develop symptoms of MG after thymectomy, just as our case 4 [1, 9].

About 30% of thymoma patients have MG, while 10–15% of MG patients have a thymoma [2]. Thymoma-associated myasthenia gravis is a paraneoplastic disease. Except for RCC, the patient in case 4 also had thymoma. In this case, the relationship between RCC and MG is difficult to establish, because MG may be more possible a paraneoplastic disease of thymoma. It is controversial that simultaneous MG with extrathymic malignancies might be one of the paraneoplastic syndromes. Extrathymic malignancies were reported to be relatively common in patients with MG, especially in the older age group [10]. In our 5/6 patients with RCC without thymoma, the average onset-age of MG was 65.8 (range from 57 to 76). Lymphoid malignancy, breast cancer and lung cancer were the most common extrathymic malignant tumors found in MG patients [10, 11]. Except for lymphoid malignancies, there was no difference in the risk of developing other malignant diseases, between the MG and comparison cohort [11]. The association between RCC and MG was only reported in one case report suggesting a paraneoplastic association [7]. There are some other case reports suggesting MG might be a paraneoplastic syndrome of other extrathymic malignancies [3–6]. Furthermore, the incidence of RCC is around 2% in this cohort of 283 MG patients, which is considerably higher than the incidence reported for the general population worldwide (6.1/100,000 in male, 3.2/100,000 in female) [12]. Our case series study provides more information of the association between MG and RCC, indicating that physicians should be aware of MG as a potential paraneoplastic syndrome that may be associated with RCC.

RCCs are commonly detected incidentally because of routine ultrasound or CT scans. The classic triad of flank pain, gross hematuria and palpable abdominal mass are found in just 6–10% of cases [13]. The presenting symptoms of RCC in these six cases including flank pain, gross hematuria and renal dysfunction, while half of them were asymptomatic. Unlike lung cancer, which is easy to find in patients with MG because they usually have chest CT to screen for thymoma [1, 5, 6]. In patients with MG, we need to fully consider the necessity of abdominal imaging to screen for other tumors including RCC.

In RCC, the most common type of paraneoplastic syndrome is the endocrine subtype. Neurological paraneoplastic syndromes are rare events in RCC [14, 15]. The mechanism of neurological paraneoplastic syndromes seems to be immunologic, associated with antibody and T cell-mediated response to antigens shared between the tumor and neural tissue. The thymus has two sources of AChRs determinants including myoid cells and medullary thymic epithelial cells that produce only unfolded subunits or fragments of AChRs [16]. Similarly, epithelial cells from the proximal convoluted tubule of the kidney, which have been proposed as the cells of origin for the two most common subtypes of RCC (clear cell RCC and papillary RCC), express functional AChRs [17, 18]. Meanwhile, all these six patients were positive for anti-AChRs antibodies. Thus, a similar mechanism may account for the production of anti-AChR antibody in the patients with RCC.

In conclusion, we described six cases of anti-AChR positive MG associated with RCC. Our cases indicate that MG as a potential paraneoplastic syndrome may be associated with RCC. Normal thymus and older age of MG onset prompt additional neoplastic screening leading to an early diagnosis of a potentially devastating cancer including RCC. A coincidental finding should not be excluded in some MG cases with RCC, further studies are needed to elucidate the relationship between MG and RCC.

## Supplementary Information

**Additional file 1: Supplementary material.**

## Data Availability

The datasets used and analyzed during the current study available from the corresponding author on reasonable request.

## Abbreviations

MG: Myasthenia gravis
AChRs: Acetylcholine receptors
RCC: Renal cell carcinoma
