# Supplementary material for: Myasthenia gravis associated with renal cell carcinoma: a paraneoplastic syndrome or just a coincidence
Source: BMC Neurol. 2021 Jul 12;21:277. doi: 10.1186/s12883-021-02311-8 (PMC8273985; doi:10.1186/s12883-021-02311-8)
Supplement: Supplementary file 1 — Additional file 1: Supplementary material. [file 12883_2021_2311_MOESM1_ESM.docx]

The clinical data of these six patients in detail:

**Case 1：**

An 83-year-old male patient had bilateral ptosis and diplopia when he was 71 years old, without symptoms such as limb weakness or dysphagia. His symptoms fluctuated throughout the day and were worse in the evening. His past medical history was unremarkable. The repetitive nerve stimulation (RNS) studies consisting low-rate or high-rate stimulation were all negative. The antibodies to the AChR, titin and ryanodine were positive. Computerized tomography (CT) of the chest was negative for the evidence of thymoma. He was diagnosed with MG (MGFA clinical classification class I) and treated with pyridostigmine, oral prednisone (60mg once a day) and azathioprine (50mg twice a day). Prednisone and azathioprine were gradually withdrawn within one year. His symptoms had improved, but he still had occasional ptosis. A right renal mass was found by abdominal ultrasound because of elevated blood creatinine at his 78-year age. Uneventful right radical nephrectomy was performed. Pathological diagnosis was grade G1 mixed type RCC including clear cell and chromophobe carcinomas, with the size of 4.1cm*3.5cm*3cm. His symptoms remained stable with occasional ptosis.

**Case 2：**

A 76-year-old female, had ptosis 8 years ago, and difficulty in swallowing 7 years ago, which needed a feeding tube. The test of RNS was positive at low-frequency stimulation and showed no increase at high-frequency stimulation. The chest CT revealed thymic hyperplasia. The anti-AChR antibody was mildly elevated. She was diagnosed with MG (MGFA class IVb), and treated with pyridostigmine, prednisolone, tacrolimus (1mg twice a day) and IVIg (0.4g/kg/d for 5 days). Her symptoms were improved temporarily, but worsened intermittently with dysphagia needing gastric tube again, and decreased proximal muscle strength (MRC 3/5), which could improve with the use of IVIg. She found a kidney mass by abdominal ultrasound without any symptoms 4 years ago. The mass progressively enlarged to the size of 4.1cm*3.1cm*4.2cm. Needle biopsy and radiofrequency ablation were performed 1 year ago. The pathological diagnosis was grade G1 clear cell RCC. Her symptoms remained unchanged after the treatment of RCC.

**Case 3：**

A 59-year-old man presented with painless gross hematuria for half a month two years ago. CT of the urinary system showed a tumor in the right kidney. He also had polycythaemia with the hemoglobin concentration elevated to 193g/L (normal range: 130-175g/L). His medical history was unremarkable. Uneventful right radical nephrectomy was performed. The pathological diagnosis was grade G1 clear cell RCC with the size of 8.5cm*7.5cm*7cm. However, he developed eyelid drooping and diplopia 1 month after the operation. After that, he also experienced shortness of breath, mild fatigable chewing and dysphagia within 1 week. The symptoms were more severe in the evening. On physical examination, his limb muscle strength was normal. His hemoglobin concentration dropped to normal. The RNS tests showed positive results at low-frequency stimulation and no significant increment at high-frequency stimulation. The anti-AChR antibody was positive, and anti-titin and ryanodine antibodies were negative. No thymoma was found by chest CT. He was diagnosed with MG (MGFA class IIb), and treated only with pyridostigmine without immunosuppressants. His symptoms resolved and the pyridostimine was eventually discontinued one year ago and he maintained a completely stable remission.

**Case 4：**

A 44-year-old man found a mass in his left kidney incidentally due to an abdominal ultrasound examination 5 years ago without any symptoms. He soon underwent a partial nephrectomy and the pathological diagnosis was clear cell RCC with the size of 3.3cm*3cm*2.7cm, most of which were grade G1 level and others were grade G2 level. Four months later, the patient had a chest CT scan due to cough, and found a thymoma in addition to bronchitis. Resection of the thymoma was performed. The pathological diagnosis was a type B2 thymoma with a size of 5.5cm*4.7cm*3.5cm. One month after thymectomy, he developed mild muscle weakness in his proximal limbs, and shortness of breath, without ptosis or diplopia. The RNS tests showed positive results at low-frequency stimulation and no significant increment at high-frequency stimulation. The anti-AChR antibody was positive, while the anti-titin and ryanodine antibodies were negative. He was diagnosed with MG (MGFA class IIa), and treated with pyridostigmine and oral prednisone (80mg once a day). His symptoms had been partially improved with an MG-ADL score of 4.

**Case 5：**

This was a male patient, presenting with left flank pain for 3 weeks when he was 51 years old. An abdominal ultrasound found a mass in the left kidney, and further abdominal CT examination considered left renal cancer with invasion of the left renal pelvic. The medical history was unremarkable except for a history of hypertension. Uneventful left radical nephrectomy was performed. The pathological diagnosis was grade G1 clear cell RCC with the size of 8.5cm*6.5cm*4cm. Because of bone metastases, radiotherapy was also performed. He had fluctuating bilateral ptosis and diplopia when he was 57 years old. He gradually developed dysarthria and dysphagia without limb muscle involvement within 3 months. An acetylcholinesterase inhibitor test was positive. RNS at a low-rate (3Hz) showed the decrement was greater than 15 percent, while no increase at high-frequency stimulation (20 Hz). The chest CT did not found thymoma. The tests for anti-AChR, titin and ryanodine antibodies were positive. He was diagnosed with MG (MGFA class IIIb) with the treatment of pyridostigmine, oral prednisone (60mg once a day) and IVIg (0.4g/kg/d for 5 days). His syndromes were improved with only occasional ptosis. However, he died of RCC metastasis at the age of 62.

**Case 6：**

A 77-year-old man developed ptosis at first, then with muscle weakness in his proximal limbs, shortness of breath and dysarthria 1 year ago. Medical history was remarkable for hypertension, diabetes, and Hashimoto's thyroiditis. The RNS tests at low or high frequencies were all negative. No thymoma was found on chest CT scans. The test for anti-AChR antibody was positive, while anti-VGCC antibody was negative. The patient was diagnosed with MG (MGFA class IVb) with an MG-ADL score of 16. After treatment with pyridostigmine, tacrolimus (1mg, twice a day) and IVIg treatment, his symptoms were relieved to an MG-ADL score of 5. During the hospitalization, a CT scan of the abdomen found a tumor in the right kidney. A radical nephrectomy was performed immediately. The pathological diagnosis was grade G1 type of clear cell RCC with the size of 5cm*4.2cm*4cm. He adjusted tacrolimus to mycophenolate mofetil because of increased blood creatinine later. During the past one year, he still had recurrent episodes of exacerbation and remission treated by IVIg. Except for persistent ptosis, he had no other symptoms until last follow-up.
